# *Angiostrongylus vasorum* infection in dogs from a cardiopulmonary dirofilariosis endemic area of Northwestern Italy: a case study and a retrospective data analysis

**DOI:** 10.1186/s12917-017-1083-7

**Published:** 2017-06-07

**Authors:** Emanuela Olivieri, Sergio Aurelio Zanzani, Alessia Libera Gazzonis, Chiara Giudice, Paola Brambilla, Isa Alberti, Stefano Romussi, Rocco Lombardo, Carlo Maria Mortellaro, Barbara Banco, Federico Maria Vanzulli, Fabrizia Veronesi, Maria Teresa Manfredi

**Affiliations:** 1 Department of Veterinary Medicine, Università degli Studi di Milano, 20133 Milan, Italy; 2 Department of Veterinary Medicine, University of Perugia, 06126 Perugia, Italy; 3 DVM, Vigevano, 27029 Pavia, Italy; 4 Department of Health, Animal Science and Food Safety, University of Milan, 20133 Milan, Italy

**Keywords:** *Angiostrongylus vasorum*, *Dirofilaria immitis*, *Crenosoma vulpis*, Dogs, Angio detect™, DiroCHEK® heartworm, Witness® *Dirofilaria*, FLOTAC

## Abstract

**Background:**

In Italy, *Angiostrongylus vasorum*, an emergent parasite, is being diagnosed in dogs from areas considered free of infection so far. As clinical signs are multiple and common to other diseases, its diagnosis can be challenging. In particular, in areas where angiostrongylosis and dirofilariosis overlap, a misleading diagnosis of cardiopulmonary dirofilariosis might occur even on the basis of possible misleading outcomes from diagnostic kits.

**Case presentation:**

Two Cavalier King Charles spaniel dogs from an Italian breeding in the Northwest were referred to a private veterinary hospital with respiratory signs. A cardiopulmonary dirofilariosis was diagnosed and the dogs treated with ivermectin, but one of them died. At necropsy, pulmonary oedema, enlargement of tracheo-bronchial lymphnodes and of cardiac right side were detected. Within the right ventricle lumen, adults of *A. vasorum* were found. All dogs from the same kennel were subjected to faecal examination by FLOTAC and Baermann’s techniques to detect *A. vasorum* first stage larvae; blood analysis by Knott’s for *Dirofilaria immitis* microfilariae, and antigenic tests for both *A. vasorum* (Angio Detect™) and *D.immitis* (DiroCHEK® Heartworm, Witness®*Dirofilaria*). The surviving dog with respiratory signs resulted positive for *A. vasorum* both at serum antigens and larval detection. Its Witness® test was low positive similarly to other four dogs from the same kennel, but false positive results due to cross reactions with *A. vasorum* were also considered. No dogs were found infected by *A. vasorum*.

Eventually, the investigation was deepened by browsing the pathological database of Veterinary Pathology Laboratories at Veterinary School of Milan University through 1998–2016, where 11 cases of angiostrongylosis were described. Two out of 11 dogs had a mixed infection with *Crenosoma vulpis*.

**Conclusion:**

The study demonstrates the need for accurate surveys to acquire proper epidemiological data on *A. vasorum* infection in Northwestern Italy and for appropriate diagnostic methods. Veterinary clinicians should be warned about the occurrence of this canine parasite and the connected risk of a misleading diagnosis, particularly in areas endemic for cardiopulmonary dirofilariosis.

## Background

*Angiostrongylus vasorum* (“French heartworm”) is a canine metastrongylid nematode of the cardiac right side and pulmonary arteries with an indirect life cycle requiring gastropods as intermediate hosts [1]. *A. vasorum* is an emergent parasite; initially notified by Serres in France in 1853 [2], it is currently widespread in tropical, subtropical and temperate regions of Africa, America and Europe with a pattern distribution in bounded endemic foci [3, 4]. Recently, frequency of this parasitic infection has increased in previously non-identified endemic areas and therefore new foci were identified [5, 6]. The apparent expansion of angiostrongylosis was ascribed to factors such as an increased disease awareness among veterinarians and researchers and the availability of proper diagnostic techniques [6, 7]. Climatic changes were also considered as one of the possible causes of the recent spread of the parasite, and studies within fox populations in Canada and European countries - that estimate a prevalence of *A. vasorum* often higher than 5–56% [4] - suggest that foxes are the most important reservoir of infection for dogs. In Italy, *A. vasorum* is being diagnosed in dogs from areas considered free of infection so far [7–11]. Diagnosis of *A. vasorum* infection is extremely challenging, since clinical signs are multiple, and its typical symptoms, such as respiratory and circulatory distress, are common to other parasitic and non parasitic diseases of dogs [4, 6]. In addition, some kits used for diagnosis of canine heartworm disease may give misleading results due to a possible cross-reaction with *A. vasorum* antigens [10, 12]. Hunting dogs as well as dogs living in suburban areas with high density of foxes are categories at elevated risk of infection [4, 13]. A few breeds, like Beagle and Cavalier King Charles spaniel appeared to be at higher risk of infection and showed severe clinical forms of angiostrongylosis [13, 14]. The main aim of the present study was to describe the occurrence of an infection of *A. vasorum* in a dog kennel located in Northwestern Italy (Lombardy region) where a few cases of canine cardiopulmonary dirofilariosis were diagnosed. A secondary aim was to perform a retrospective analysis of *A. vasorum* infections recorded in the pathological database of the Veterinary Pathology Laboratories at Veterinary School of Milan University in order to update the epidemiological data that are very scarce for this area.

## Case presentation

**Fig. 1:**
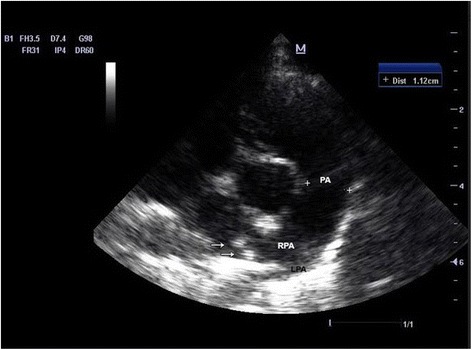
Two-dimensional echocardiogram right parasternal short axis view. Notice the punctate hyperechoic areas (*white* arrows) inside the right trunk of right pulmonary artery (RPA). LPA = left pulmonary artery. PA = pulmonary artery

In February 2015, a two-year-old female of Cavalier King Charles spaniel (CKCS) (Table 1; Dog-ID = 10) was referred for apathy and respiratory distress in a private veterinary hospital. At clinical examination, the dog showed poor general condition, dehydration, severe dyspnoea and profuse hemoptysis. Radiographic examination of the thorax revealed a generalised interstitial and alveolar pattern. Echocardiography showed dilatation of the main pulmonary artery and its right branch was evidenced, and hyperechoic areas were also observed (Fig. 1). Witness® *Dirofilaria* test (Zoetis, Florham Park, NJ, USA) for the detection of circulating antigens released by adults of *D. immitis* showed low positive results*,* and Knott’s test for the detection of the microfilariae was negative. Based on these results, the veterinarian diagnosed a cardiopulmonary dirofilariosis and the dog was subcutaneously treated with ivermectin (IVOMEC®, Merial Italia, Padova, Italy) at off-label dosage (300 μg/kg). The dog health condition improved quickly after the first medication; then, a following treatment with ivermectin–pyrantel pamoate (Cardotek Plus®, Merial Italia, Padova, Italy) at a dose of 6 μg/kg (ivermectin) and 5 mg/kg (pyrantel pamoate) per os once every 15 days was administered for 6 months. A few days after the first dog underwent clinical examination, an 8-month-old female (Table 1; Dog-ID = 20) from the same breeding was referred with similar clinical signs and diagnosed with a heartworm disease. As in the previous case, the Witness® *Dirofilaria* was low positive. The prescribed treatment was ivermectin–pyrantel pamoate (Cardotek Plus®, Merial, Italia, Padova, Italy) at a dose of 6 μg/kg (ivermectin) and 5 mg/kg (pyrantel pamoate) per os once every 15 days for 6 months and doxycycline (RONAXAN®, Merial, Italia, Padova, Italy) at a dose of 10 mg/kg per os daily for 30 days, but the dog died after two days. A complete necropsy performed within 24 h from death revealed that the dog was in good body condition. Main pathological findings were severe, diffuse pulmonary oedema with large, locally extensive areas of consolidation of the lung parenchyma; diffuse, severe enlargement of tracheo-bronchial lymphnodes and marked enlargement of the heart with right atrial dilation and right ventricular hypertrophy. Within the right ventricle lumen, numerous slender and approximately 18–25 mm long nematodes were detected.

**Table 1:** Comparative results of diagnostic tests for *Angiostrongylus vasorum* infection in all dogs from a kennel in northwestern Italy

Dog-ID	Breed^a^	Sex^b^	Age (months)	Origin^c^	FLOTAC	Baermann test^d^	Knott test^d^	Witness®^d^	DiroCHEK®^d^	Angio Detect™^d^
1	CKCS	F	89	I (France)	N ^f^	N	N	N	N	N
2	CKCS	M	90	I (France)	N	N	N	N	N	N
3	CKCS	F	63	BB	N	N	N	LP	N	N
4	CKCS	M	45	I (France)	N	N	N	LP	N	N
5	CKCS	F	37	I (France)	N	N	N	N	N	N
6	CKCS	F	31	BB	N	N	N	N	N	N
7	CKCS	F	31	BB	N	N	N	N	N	N
8	CKCS	F	19	BB	N	N	N	N	N	N
9	CKCS	F	22	I (France)	N	N	N	LP	N	N
10	CKCS	F	24	I (France)	L1 of *A. vasorum*	L1 of *A. vasorum*	N	LP	N	P ^h^
11	CKCS	F	15	I (France)	N	N	N	LP	N	N
12	CKCS	M	16	I (France)	N	N	N	N	N	N
13	CKCS	M	14	I (France)	N	N	N	N	N	N
14	CKCS	F	6	I (France)	N	N	N	N	N	N
15	CKCS	F	52	BB	N	N	N	N	N	N
16	CKCS	F	15	BB	N	N	N	N	N	N
17	BS	M	22	I (France)	N	N	N	N	N	N
18	BS	M	22	I (France)	N	N	N	N	N	N
19	BS	M	20	I (France)	N	N	N	N	N	N
20*	CKCS	F	8	I (France)	ND	ND	ND	ND	ND	ND

^a^ *CKCS* = Cavalier King Charles spaniel, *BS* = Belgian Shepherd; ^b^:*F* = female, *M* = male; ^b^: *I* = Imported, *BB* = Born in the breeding; ^e^, ^c^:*N* = negative, *P* = positive, *LP* = low positive, *ND* = not determined; *: angiostrongylosis diagnosed at necropsy

**Fig. 2:**
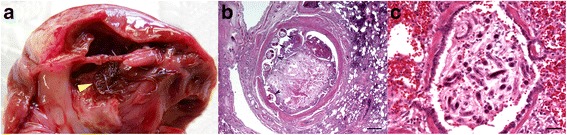
**a**) detail of the opened right heart chamber: a large mass of adult nematodes is visible in the ventricle lumen (arrowhead); **b**) histological section of lung showing a large thrombus within an arterial vessel lumen. Numerous cross sections of adult nematodes are present near the vessel wall (arrowheads). Haematoxylin and Eosin, bar 500 μm; **c**) histological section of lung showing numerous cross- and longitudinal sections of nematodal larvae filling the lumen of a bronchiole. Haematoxylin and Eosin, bar 50 μm

Worms were collected, fixed in 100% alcohol and submitted for parasitological examination; they were morphologically identified as adults of *A. vasorum* according to the taxonomical keys [1, 15] (Fig. 2). At necropsy, cytological samples obtained from the lungs revealed the presence of numerous larvae of *A. vasorum* within the pulmonary parenchyma (Fig. 2).

Histologically, lungs were characterized by multifocal, acute haemorrhages, diffuse alveolar oedema and severe, diffuse granulomatous pneumonia, with myriad intralesional nematode eggs and larvae. Eggs were thin-walled, ovoid, measured 50–60 μm in diameter and contained either a morula or a larva. Sections of adult worms, partially surrounded by large thrombi, were also frequently detected within the lumen of pulmonary arteries. Innumerable larvae were also present within trachea-bronchial lymphnodes, infiltrating and distending cortical and medullary sinuses.

**Fig. 3:**
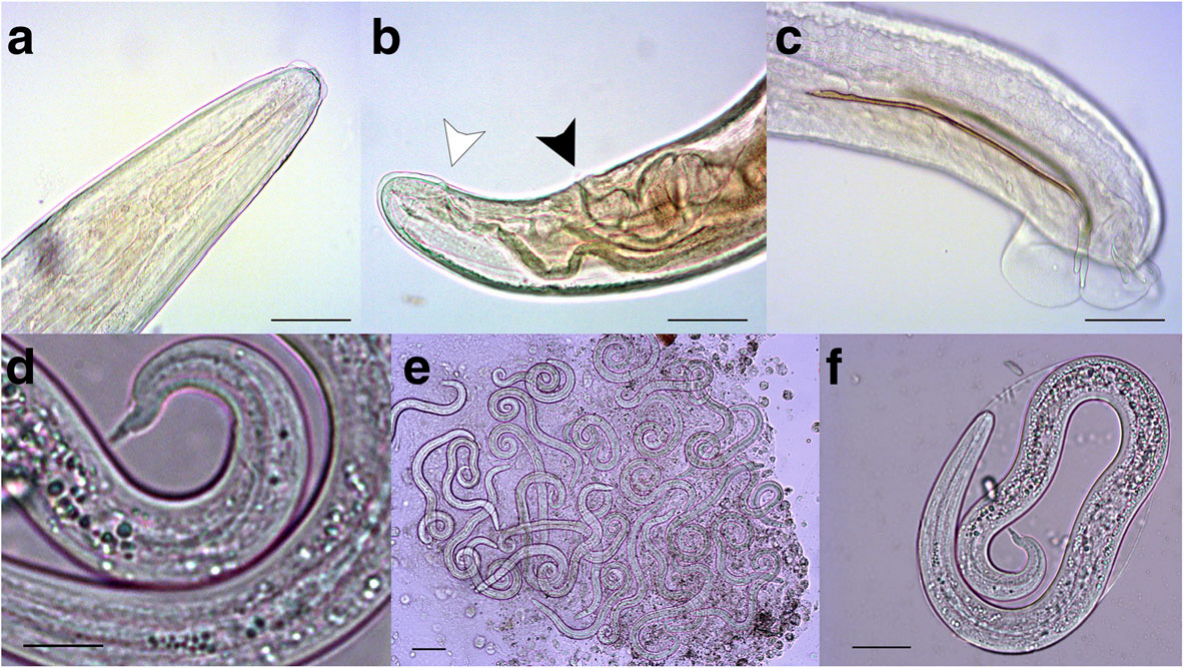
*Angiostrongylus vasorum* from dogs: **a**) details of anterior extremity of an adult worm showing esophagus, scale bar 100 μm; **b**) details of adult female caudal extremity, presenting vulva (black arrowhead) and anus (white arrowhead), scale bar 100 μm; **c**) male caudal extremity showing the copulatory bursa with spicules, scale bar 100 μm; **d**) Baermann test: first-stage larva of *A. vasorum*, scale bar 20 μm; **e**) numerous larvae recovered from the bronchoalveolar lavage (BAL) incorporated in strands of mucus, scale bar 50 μm; **f**) eggs containing a coiled larva, recovered from BAL, scale bar 20 μm

Considering a definitive diagnosis of *A. vasorum* on the dog ((Table 1; Dog-ID = 20), other dogs from the same kennel were also investigated to evaluate the presence of the parasite. Out of nineteen dogs there sheltered, 16 were CKCS and three were Belgian shepherds. The majority of them originated from France (Table 1). All dogs were subjected to faecal examination using Baermann’s and the FLOTAC techniques, the last having an analytic sensitivity of two larvae per gram (LPG) of faeces; a zinc sulphate-based solution (specific gravity = 1.360) was used to detect first-stage larvae (L1) of *A. vasorum* [16, 17]. A rapid immunochromatographic test (Angio Detect™, IDEXX Laboratories, Westbrook, Maine, USA) with sensitivity 84.6% (95% C.I. 69.5–94.1%) and specificity 100% (95% C.I. 97.6–100%) able to reveal circulating *A. vasorum* antigens was run to verify a potential infection [18] All dogs were tested for microfilariae by Knott’s assay [19] and to evaluate a potential cross-reaction they were also analysed for serological detection of circulating *D. immitis* antigens, both by Enzyme Linked Immuno Assay (ELISA) (DiroCHEK® Heartworm antigen test kit, Synbiotics, San Diego, USA) and rapid immunomigration (Witness *Dirofilaria*®, Zoetis, Florham Park, NJ, USA) tests. All kits were used following the manufacturer instructions. Results of these analyses are reported in Table 1. The samples belonging to the dog initially referred for respiratory distress (Table 1; Dog-ID = 10) were positive both for *A. vasorum* antigens and L1.The several L1 of *A. vasorum* isolated by Baermann’s technique were 340–380 μm long with a typical tail (Fig. 3). Knott’s test and DiroCHEK®ELISA were both negative, but the Witness® test was low positive. Four other dogs had a low positive result to this test but were negative to DiroCHEK® ELISA and to other performed tests (faecal examination, Knott’s test, Angio Detect™)(Table 1).

The entire archive of the Veterinary Pathology Laboratories at Veterinary School of Milan University (VPL) was browsed through 1998–2016 to collect data on diagnosed *A. vasorum* infections at necropsy or at faecal examination. Overall, 11 cases of angiostrongylosis were discovered (Table 2). In the studied period an overall of 2521 dogs had undergone necropsy, 1820 dogs (1998–2009) and 701 dogs (2010–2016), respectively. No dogs were found positive for *A. vasorum* infection in the first mentioned period (0/1820; 0%, 95% CI: 0%–0.21%), whereas five dogs were found positive in the second period (5/701, 0.71%, 95% CI: 0.3%–1.65%). Pearson’s Chi-square test showed significant difference in the prevalence of *A. vasorum* infection in the two periods (*p*-value = 0.00031). Further, all results of the Baermann tests present in the archive were considered; particularly, data on 82 Baermann tests (19.57%, 95% CI: 16.05%–23.64%) out of 419 dog’s faecal samples sent to parasitological service of the VPL in the period 2011–2016 were found. All tests were required by clinicians of the veterinary hospital following dog clinical examinations and thanks to the assent of the dogs’ owners. The percentage of required Baermann tests through the years was evaluated with Pearson’s Chi-square test and resulted not significant (*p*-value = 0.999). Thus, apparently, from 2011 to 2016 cardiopulmonary parasites had not received increasing attention by clinicians, who probably submitted samples for Baermann test just from clinically suspected dogs. Data on other diagnostic analysis, if present in the archive, were added (Table 2).

**Table 2:** Cases of angiostrongylosis discovered browsing the pathological database of Veterinary Pathology Laboratories through 1998–2016 at Veterinary School of Milan University (Northwestern Italy)

Dog ID	Date (month-year)	Breed, age (months), sex^a, b^	Clinical signs	Outcome	Postmortem finding^b^	Angio Detect™ ^b,c^	Isolation of first stage larvae^b,c^
1	February 2016	Jack Russel, 24, M	Respiratory distress	good	ND	P	P(Bal)*
2	March 2015	Cao de Agua Portuguese, 24, M	Respiratory distress	good	ND	ND	P (Fl, Baermann test, Bal)
3	December 2014	Mixed-breed, 48, F	Fever	good	ND	P	N (Baermann test)
4	June 2014	Maltese, 12, M	Respiratory distress	good	ND	P	N (Baermann test)
5	April 2014	Mixed-breed, 144, M	Respiratory distress	good	ND	P	N (Baermann test)
6	March 2014	Mixed-breed, 12, F	Respiratory distress	died	pulmonary oedema and hyperaemia adult worm in pulmonary artery	ND	ND
7	March 2014	Springer spaniel, ND., F	Respiratory distress	died	pneumothorax, pneumomediastinum severe, diffuse, pulmonary oedema - severe, diffuse necrotizing haemorrhagic pneumonia with many eggs and larvae right atrial and ventricular dilatation larvae infiltrated medullary sinuses of trachea-bronchial lymph-nodes	ND	ND
8	February 2014	Italian hound, 96, F	Tetraparesis	good	ND	ND	P (Fl, Baermann test, Bal)*
9	January 2014	Lurcher, 84, F	Respiratory distress	good	ND	P	N
10	January 2014	Mixed-breed, 12, M	Ataxia	good	ND	P	P (Baermann test)
11	February 2010	Rhodesian Ridgeback, 8, F	Coagulation disorders and Respiratory distress	died	bleeding from the nasal cavities and all the vessels sectioned extensive areas of pulmonary oedema and haemorrhages section of larvae in kidney and lungs	ND	ND

^a^: *F* = female, *M* = male; ^b^: *N* = negative, *P* = positive, ^b^: *ND* = not determined; ^c^: *Fl* = FLOTAC, *Bal* = Bronchoalveolar lavage, *mixed infection, first stage larvae of *Angiostrongylus vasorum* and *Crenosoma vulpis* occurred

As regards their provenience, ten of the infected dogs were from districts of four provinces of Lombardy whereas one was a dog (case 6) recently adopted from a shelter in Sardinia. Two out of 11 dogs showed a mixed infection with *Crenosoma vulpis*.

The current and other recent reports provide important evidence that in northern Italy *A. vasorum* infection has spread more than expected [10, 20]. Particularly, the cases of canine angiostrongylosis reported here emphasize that moving dogs within different areas could pose a risk of infection. In fact, two cases of angiostrongylosis were detected in show dogs belonging to a kennel of Northwestern Italy but originally coming from two French kennels situated in a region endemic for *A. vasorum,* where they could have acquired the infection [21]. However, even though these dogs were not usually walked to public green areas, their age together with the short pre-patent period of the parasite can support an autochthonous infection. Indeed, a few cases of angiostrongylosis had been already reported in dogs from the same area [7, 10]. Further, one out of 11 cases from the pathological database of the VPL was a dog from another region of Italy where angiostrongylosis is present [22]. Data from this study also support the hypothesis that Northwestern Italy could be a compatible territory for parasite transmission by eco-climatic index with focal areas of high suitability [23]. Moreover, foxes seem to be highly spread in the studied area (http://www.cartografia.regione.lombardia.it/agrinet/natura_volpe_RL.htm) even though none of the parasitological researches carried out here was targeting *A. vasorum* infection in these carnivores [24]. In addition, the present study underscores both the complexity and difficulty of diagnosis related to this parasitic infection [25, 26]. In fact, angiostrongylosis was not readily recognized in the two infected CKCS because of a few clinical signs overlapping cardiopulmonary dirofilariosis and a slight positive reaction to the antigenic test for *D. immitis*. It is likely for some veterinarians to be mistaken about diagnosis of angiostrongylosis mainly when they work in an area considered hyperendemic for dirofilariosis, such as the one pertaining to the kennel under consideration, which is confirmed by the present study. Particularly, low positive reactions for *D. immitis* at Witness® test occurred with sera of dogs infected with *A. vasorum*, while previous studies already showed the existence of cross-reaction with DiroCHECK test [12] and with IDEXX 4Dx Plus®Test [10]. Thus, it is evident that, in geographical areas where both parasites are present, antigenic tests for the detection of *D. immitis* may produce false positive results; therefore, in dogs with a suspected heartworm infection, the use of specific diagnostic tools for both *D. immitis* and *A. vasorum* is highly recommended. The mixed infection (*A. vasorum* and *C. vulpis*) found in two dogs highlights the need for an appropriate identification of L1 recovered in faecal samples or bronchoalveolar lavage. Crenosomosis has been recognised as an important cause of chronic respiratory disease in dogs in Europe and it is often mistaken with allergic respiratory disease [27]. Dogs exposed to *C. vulpis* are also at risk of *A. vasorum* infection due to partial overlapping of intermediate hosts, and the occurrence of a mixed infection should be considered both for not misdiagnosis and proper treatment [28]. As regards the occurrence of a severe clinical form in CKCS, it was previously reported that CKCS are frequently infected by *A. vasorum* and a few authors suggested that this breed may be at an increasing risk of the disease [14]. Considering that CKCS and other surveyed breeds were exposed to the same risk factors, the cited authors hypothesized that the high incidence of severe clinical forms in CKCS could be related to a defect of their immune response to infection by *A. vasorum* [14].

## Conclusion

The findings of the present study demonstrate the need for accurate surveys to acquire proper epidemiological data on *A. vasorum* infection in Southern Europe given the relevance of the disease in dogs and the suitability of the area to this nematode life-cycle. It also urges to increase veterinaries awareness about the occurrence of this canine parasite still largely underestimated and the risks of an incorrect diagnosis particularly in areas endemic for cardiopulmonary dirofilariosis [29, 30].

## Abbreviations

CKCS: Cavalier King Charles spaniel
VPL: Veterinary Pathology Laboratories
